# Cost-Effectiveness of [^177^Lu]Lu-DOTATATE for the Treatment of Newly Diagnosed Advanced Gastroenteropancreatic Neuroendocrine Tumors: An Analysis Based on Results of the NETTER-2 Trial

**DOI:** 10.2967/jnumed.124.269416

**Published:** 2025-07

**Authors:** Adrien Holzgreve, Lena M. Unterrainer, Maximilian Tiling, Nabeel Mansour, Christine Spitzweg, Matthias Brendel, Jens Ricke, Marcus Unterrainer, Wolfgang G. Kunz, Dirk Mehrens

**Affiliations:** 1 Department of Nuclear Medicine, LMU University Hospital, LMU Munich, Munich, Germany;; 2 Ahmanson Translational Theranostics Division, David Geffen School of Medicine at UCLA, Los Angeles, California;; 3 Bavarian Cancer Research Center, Partner Site Munich, Munich, Germany;; 4 Department of Radiology, LMU University Hospital, LMU Munich, Munich, Germany;; 5 Department of Internal Medicine IV, LMU University Hospital, LMU Munich, Munich, Germany;; 6 DZNE–German Center for Neurodegenerative Diseases, Munich, Germany;; 7 Munich Cluster for Systems Neurology (SyNergy), University of Munich, Munich, Germany;; 8 German Cancer Consortium, Partner Site Munich, a partnership between DKFZ and LMU, Munich, Germany; and; 9 Die Radiologie, Munich, Germany

**Keywords:** neuroendocrine, oncology, radionuclide therapy, cost-effectiveness analysis, GEPNET, NETTER-2

## Abstract

The recently published results of the NETTER-2 trial suggest the use of [^177^Lu]Lu-DOTATATE as a new standard of care in first-line therapy of patients with grade 2 or 3, well-differentiated, advanced gastroenteropancreatic neuroendocrine tumors. The NETTER-2 trial found superior median progression-free survival (22.8 mo vs. 8.5 mo) and similar adverse events and quality-of-life measures in patients treated with [^177^Lu]Lu-DOTATATE compared with octreotide long-acting release (LAR) alone. As [^177^Lu]Lu-DOTATATE therapy is associated with higher costs, we compared the cost-effectiveness of [^177^Lu]Lu-DOTATATE with that of octreotide LAR in this setting. **Methods:** A partitioned survival model was established for the trial duration of 36 mo as well as a lifetime horizon of 20 y. Progression-free survival and treatment regimens for each patient group were derived from the NETTER-2 trial. Information on overall survival as well as utilities for health states and utilities and costs of adverse events were obtained from the literature. Information on treatment costs was obtained from the Centers for Medicare and Medicaid Services. The willingness-to-pay threshold was set to $100,000 per quality-adjusted life-year (QALY). **Results:** [^177^Lu]Lu-DOTATATE was cost-effective during the trial duration, with an incremental QALY of 0.13 at a slightly higher cost ($8,931), leading to an incremental cost-effectiveness ratio of $66,761/QALY. For the lifetime analysis, [^177^Lu]Lu-DOTATATE dominated treatment with octreotide LAR. However, deterministic sensitivity analysis revealed that the incremental cost-effectiveness ratio was strongly influenced by the percentage of patients treated with [^177^Lu]Lu-DOTATATE after disease progression as well as number of cycles of [^177^Lu]Lu-DOTATATE they received after progression. In the probabilistic sensitivity analysis using Monte Carlo simulation with 10,000 iterations, [^177^Lu]Lu-DOTATATE proved to be the most cost-effective strategy for 64% of Monte Carlo iterations over the trial duration. **Conclusion:** [^177^Lu]Lu-DOTATATE is cost-effective as a first-line treatment for patients with grade 2 or 3, well-differentiated, advanced gastroenteropancreatic neuroendocrine tumors.

## Visual Abstract

**Figure:**
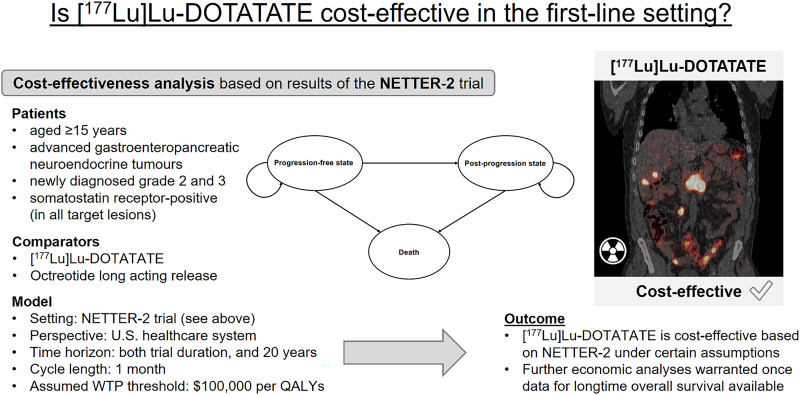

Gastroenteropancreatic neuroendocrine tumors (GEP-NETs) are rising in both incidence and prevalence (1*,*^2^). In 2017, grade 3, well-differentiated GEP-NETs were classified as an independent tumor entity (3). The combination of [^177^Lu]Lu-DOTATATE and low-dose octreotide long-acting release (LAR) was shown to be superior to high-dose octreotide LAR alone for lower-grade, midgut NETs in the NETTER-1 trial (4). The open-label, randomized, parallel-group, superiority, phase 3 NETTER-2 trial compared the effect of [^177^Lu]Lu-DOTATATE and low-dose octreotide LAR with high-dose octreotide LAR in well-differentiated, grade 2 or 3, advanced GEP-NETs (5). Results of the NETTER-2 trial found superior median progression-free survival (PFS) (22.8 vs. 8.5 mo) and similar adverse events and quality-of-life measures in patients treated with [^177^Lu]Lu-DOTATATE compared with octreotide LAR alone. [^177^Lu]Lu-DOTATATE has already been included in guidelines as a second-line systemic option for suitable patients with higher grade GEP-NETs (6). However, the NETTER-2 trial suggests the use of [^177^Lu]Lu-DOTATATE as a first-line treatment option for this specific patient group.

As [^177^Lu]Lu-DOTATATE therapy is associated with higher costs, the aim of this study was to evaluate the cost-effectiveness of [^177^Lu]Lu-DOTATATE for patients with higher-grade, well-differentiated GEP-NETs based on data from the NETTER-2 trial.

## MATERIALS AND METHODS

### Study Population

The study population of the NETTER-2 trial (NCT03972488) consisted of 226 individuals (Table 1) with newly diagnosed (within the past 6 mo), advanced, somatostatin receptor–positive, well-differentiated, grade 2 or 3 GEP-NETs with a Ki-67 proliferation index of 10%–55%. Patients were also ineligible if they had received any previous peptide receptor radionuclide therapy (PRRT), hepatic artery embolization, or radiofrequency ablation for GEP-NETs. Previous systemic therapy for GEP-NETs was not allowed unless it was administered for less than 1 mo and not within 12 weeks before randomization.

**TABLE 1.** Patient Characteristics per Study Group in the NETTER-2 Trial

Characteristic	[^177^Lu]Lu-DOTATATE group	Octreotide LAR group
Age (y)	61	60
Women (%)	46	47
Grade 2 GEP-NET (%)	66	64
Metastases (%)	>99	99

Age expressed as median.

Patients were randomized in a 2:1 ratio to receive [^177^Lu]Lu-DOTATATE (7.4 GBq) and 30 mg of octreotide LAR every 8 wk for a maximum of 4 cycles (88% completed 4 cycles, and 93% completed 3 cycles) and monthly treatment with 30 mg of octreotide thereafter (PRRT group) or 60 mg of octreotide LAR every 4 wk (control group). After disease progression, 19% of patients in the PRRT group underwent retreatment with [^177^Lu]Lu-DOTATATE (7.4 GBq) for 2–4 cycles every 8 wk. Retreatment with octreotide LAR in this group was based on the discretion of the investigator. Of the patients in the control group whose disease progressed, 67% chose to receive [^177^Lu]Lu-DOTATATE (7.4 GBq) combined with 30 mg of octreotide LAR every 8 wk for 4 cycles and monthly treatment with 30 mg of octreotide LAR thereafter.

### Model Structure

**FIGURE 1.**
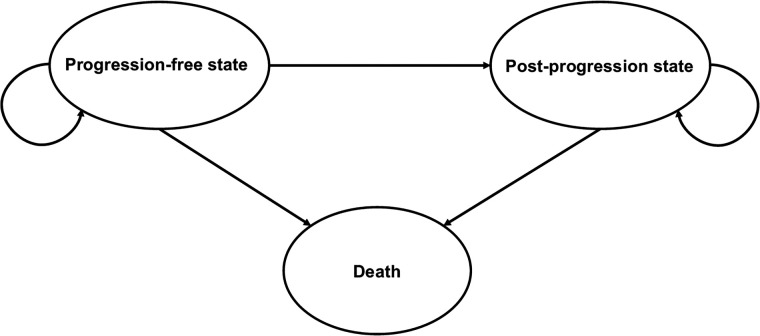
Decision model. Health state transition diagram for modeling costs and effectiveness. All patients started in progression-free state. During each cycle, patients were classified as staying in progression-free state, having transitioned to postprogression state, or having transitioned to terminal state (death).

A partitioned survival analysis model was developed using decision-analytic software (TreeAge Pro Health Care, version 2024; TreeAge Software). Patients were assigned to the PRRT group or control group. Cycle length was set to 1 mo to depict slight changes in survival and their effect on the analysis. All patients started in the progression-free state. During each cycle, patients were classified as staying in a progression-free state, having transitioned to a postprogression state, or having transitioned to a terminal state (death) (Fig. 1).

The analysis was performed according to the Consolidated Health Economic Evaluation Reporting Standards (Supplemental Table 1) (7).

### Model Input Parameters

#### Survival

PFS for both study groups was derived from the Kaplan–Meier data provided by the NETTER-2 trial for the study duration. For the lifetime horizon analysis, PFS data were extracted from the Kaplan–Meier survival curves by reconstruction of individual patient data (8). Subsequently, parametric distributions were fitted to individual patient data using statistical programming language R (version 4.2.1; R Core Team). Best-fit analysis was conducted using the Akaike information criterion, log-likelihood test, and visual control (Supplemental Fig. 1; supplemental materials are available at http://jnm.snmjournals.org).

As no long-term data for survival were available for the NETTER-2 trial at the time of our analysis, we used data from the literature to simulate overall survival (OS) for the lifetime horizon analysis of 20 y (98.1% of patients deceased in our model), according to a previous cost-effectiveness study of the NETTER-1 trial results (4*,*^9^). As no difference in OS was noted between the PRRT and octreotide group in the NETTER-2 trial, the assumption was made that long-term survival was also comparable between groups. This assumption was supported by the results of the NETTER-1 trial. Similar to the NETTER-2 trial, NETTER-1 included patients with locally advanced or metastatic, well-differentiated, somatostatin receptor–positive midgut neuroendocrine tumors. However, NETTER-1 specified a Ki-67 proliferation index of 20% or less as an inclusion criterion, whereas NETTER-2 enrolled patients using a specified Ki-67 proliferation index of 10%–55%.

The heterogeneity of both study groups was accounted for in terms of proportion of grade 2 and 3 NETs in the NETTER-2 trial (Table 1). Data extraction and fitting of parametric distributions were performed in the same manner as mentioned above. Detailed information regarding the computed parameters for each distribution is shown in Table 2.

**TABLE 2.** Detailed Input Parameters

Input parameter	Base case value	Range for sensitivity analysis*	Distribution
Initial probability (5)			
Progression-free disease	1		
Progressive disease	0		
Death	0		
Survival probability			
PPS GEP-NET (9)			
Grade 2, stage 4	Shape, 0.9628; scale, 64.1457	Shape, 0.8182–1.1328; scale 51.6802–79.6178	Weibull
Grade 3, stage 4^†^	2.0057 ± 1.1133	1.7875–2.2239 ± 0.9692–1.2788	Lognormal
PFS			
[^177^Lu]Lu-DOTATATE^†^ (5)	3.2597 ± 0.9401	3.057–3.4624 ± 0.7794–1.134	Lognormal
PFS octreotide^†^(5)	2.2833 ± 1.074	2.0671–2.4994 ± 0.9134–1.2628	Lognormal
Health care cost			
[^177^Lu]Lu-DOTATATE ($/cycle) (10)	57,617	54,736–60,498	γ
Patient retreatment with [^177^Lu]Lu-DOTATATE after progression (%)^†^ (5)			
[^177^Lu]Lu-DOTATATE	0.19	0.18–0.20	β
Octreotide LAR	0.67	0.64–0.70	β
Standard care ($/mo)			
Octreotide LAR 30 mg (HCPCS)	6,549	6,222–6,876	γ
Octreotide LAR 60 mg (11)	13,098	12,443–13,753	γ
End of life (12)	18,025	17,124–18,926	γ
Utilities			
PFS (15)	0.771	0.731–0.810	β
PPS (15)	0.612	0.564–0.659	β
Adverse events (grade 3 or 4)			
Disutility per mo (5*,*^16^)			
[^177^Lu]Lu-DOTATATE	0.01	0.008–0.012	β
Octreotide LAR	0.00287	0.0025–0.003	β
Treatment cost ($/mo) (13)			
[^177^Lu]Lu-DOTATATE	3,341	3,174–3,508	γ
Octreotide LAR	2,515	2,389–2,641	γ

* Determined by 95% CI of initial probabilities and costs.

† Median ± SD.

HCPCS = Healthcare Common Procedure Coding System.

#### Costs

This analysis was performed from a U.S. health care perspective. Standard treatment costs for PFS and postprogression survival (PPS) were derived from Medicare reimbursement data (10*,*^11^). Costs for end-of-life care and grade 3 and 4 adverse events were derived from the literature (12*,*^13^). An annual discount of 3% for costs was applied in the analysis. All costs were adjusted to 2024 prices using the U.S. Consumer Price Index (14).

#### Utilities

Treatment effectiveness was measured by quality-adjusted life-years (QALYs), calculated by multiplying years spent in progression-free and postprogression states by assigned utility weights. Utility weights for survival in the progression-free and postprogression states were derived from the literature (15). As utility data for the NETTER-2 trial were not yet available, we used the utility weights established for the NETTER-1 trial, as both study populations overlapped and study design was congruent. Frequencies for disutilities of grade 3 and 4 adverse events were derived from the NETTER-2 trial. Adverse events that occurred in more than 5% of patients were included. Values for disutilities were derived from the literature and accounted for during the treatment duration (16). Table 2 provides an overview of the input parameters. An annual discount of 3% for utilities was applied in the analysis.

### Outcomes

#### Cost-Effectiveness Analysis

The costs, effectiveness (measured in QALYs), incremental cost-effectiveness ratios (ICERs), and net monetary benefits of [^177^Lu]Lu-DOTATATE and high-dose octreotide LAR were compared over the trial duration and lifetime horizon. Incremental costs, utilities, and ICERs were calculated for each patient subgroup separately. The willingness-to-pay (WTP) threshold was set to $100,000/QALY (17). This threshold value classifies medical services in terms of their eligibility for reimbursement (18). As no data were available concerning the number of treatment cycles of PRRT administered in the [^177^Lu]Lu-DOTATATE group after disease progression, 4 cycles were chosen for analysis purposes.

#### Sensitivity Analyses

Comprehensive deterministic and probabilistic sensitivity analyses were used to evaluate the robustness of the model. Deterministic 1-way sensitivity analysis was performed to identify variables that significantly influenced model outcomes. The ranges for deterministic sensitivity analysis were determined by the 95% CI of the initial probabilities and costs. A range of 0–8 (with 8 equating to double treatment) were assumed for [^177^Lu]Lu-DOTATATE treatment. Moreover, probabilistic sensitivity analyses allow simultaneous alteration of multiple input parameters using distributions according to probability density functions for second-order Monte Carlo simulation runs (*n* = 10,000) (19). Furthermore, a cost-effectiveness acceptability curve was generated from the Monte Carlo iterations at varying WTP thresholds.

The input parameters were assigned appropriate distributions (Table 2). Utilities were varied with a β-distribution. Treatment costs were modeled with a γ-distribution. β-distributions were used for disutilities as well as utilities for survival in the progression-free and postprogression states.

To further account for uncertainty in our long-term analysis of 20 y, additional analyses were performed for OS by calculating a range of hazard ratios (0.10, 0.25, 0.50, 0.75, 1.25, 1.50, 1.75, and 2.00) for both parametric distributions used for grade 2 and 3 tumors in the analysis. No difference in survival between groups was assumed. Base case analyses were performed over the trial duration and lifetime horizon (Supplemental Table 2; Supplemental Fig. 2).

## RESULTS

### Base Case Analyses

In the base case analyses, [^177^Lu]Lu-DOTATATE was associated with an average of 1.5 QALYs over the trial duration, compared with 1.4 QALYs for octreotide LAR. The average treatment cost was $469,578 for the [^177^Lu]Lu-DOTATATE group, $8,931 higher than those for the octreotide LAR group ($460,647), resulting in an ICER of $66,761 at a net monetary benefit of $160,991. When analyzing costs and effectiveness over a lifetime horizon of 20 y, [^177^Lu]Lu-DOTATATE dominated treatment with octreotide LAR, with higher effectiveness (2.69 QALYs vs. 2.43 QALYs) and a slightly lower cost ($618,827 vs. $621,142).

Results for base case analyses are summarized in Table 3. For additional base case analysis of hazard ratios for OS of 0.1–2.0, the ICER stayed below the WTP threshold of $100,000/QALY over the trial duration (range, $66,830/QALY to $61,431/QALY) and lifetime horizon, with PRRT remaining the dominant strategy throughout (Supplemental Table 2).

**TABLE 3.** Base Case Analysis

Patient group	Cost ($)	IC ($)	Effect (QALY)	IE (QALY)	NMB ($)	ICER ($/QALY)	Acceptability at WTP/$100,000 (%)
Trial duration*							
[^177^Lu]Lu-DOTATATE	469,578	8,931	1.54	0.13	−160,991	66,761	64
Octreotide LAR	460,647		1.41		−178,812		36
Lifetime horizon^†^							
[^177^Lu]Lu-DOTATATE	618,827		2.69		−80,347		
Octreotide LAR	621,142	2,315	2.43	−0.26	−135,502	dominated	

* 36 mo.

† 20 y.

IC = incremental cost; IE = incremental effectiveness; NMB = net monetary benefit.

### Deterministic Sensitivity Analysis

**FIGURE 2.**
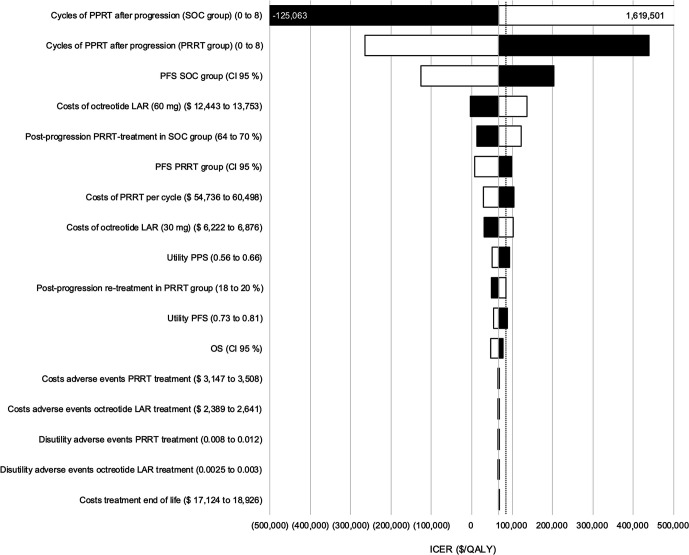
Tornado diagram for deterministic sensitivity analysis. Number of cycles of [^177^Lu]Lu-DOTATATE after progression had strongest cumulative impact on ICER followed by PFS of control (standard of care [SOC]) group, costs for octreotide LAR, and proportion of patients in control group receiving [^177^Lu]Lu-DOTATATE after progression. Black bars indicate changes based on lower bound of parameter variation. White bars indicate upper bound of respective parameter. Dotted line represents WTP threshold of $100,000/QALY.

Deterministic sensitivity analysis revealed that the number of cycles of [^177^Lu]Lu-DOTATATE after disease progression had the strongest cumulative effect on the ICER, followed by PFS of the control group, cost of octreotide LAR, and proportion of patients in the control group receiving PRRT after disease progression exceeding the WTP threshold of $100,000/QALY. PFS in the [^177^Lu]Lu-DOTATATE group, costs of [^177^Lu]Lu-DOTATATE, and costs of octreotide LAR concomitant to [^177^Lu]Lu-DOTATATE treatment affected the ICER under the chosen range, surpassing the WTP threshold. Parameters for parametric survival in the octreotide group for PFS partially led to an increase of the ICER above the WTP threshold. All other parameters in the given ranges had no significant effect on the ICER (Fig. 2).

### Probabilistic Sensitivity Analysis

**FIGURE 3.**
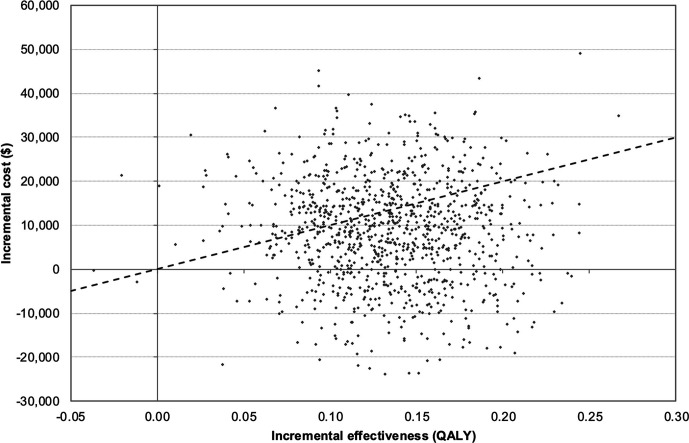
Cost-effectiveness planes of incremental costs and incremental effectiveness of [^177^Lu]Lu-DOTATATE vs. octreotide LAR are shown for probabilistic sensitivity analysis. Each dot represents one simulation run. Dashed line indicates WTP threshold of $100,000/QALY. Dots below this line are considered cost-effective simulation runs.

Probabilistic sensitivity analysis was performed for the base case scenario over 36 mo, resulting in 64.47% of the calculations to be cost-effective when applying a WTP threshold of $100,000/QALY (Fig. 3). The distribution of calculations in each quadrant was as follows: lower right (dominant), 22.8%; upper right, 76.9%; lower left, 2.0%; and upper right (dominated), 1.0%.

In the cost-effectiveness acceptability curve, octreotide LAR remained the cost-effective option until a WTP of $51,000/QALY was reached. At WTP thresholds above this value, [^177^Lu]Lu-DOTATATE became the more likely cost-effective treatment (Supplemental Fig. 3).

## DISCUSSION

The NETTER-2 trial was the first phase 3 trial to investigate [^177^Lu]Lu-DOTATATE treatment in newly diagnosed patients with advanced, grade 2 or 3 GEP-NETs and the first phase 3 trial of PRRT in any metastatic first-line treatment setting. This is particularly important, as no first-line treatment has been established for this specific tumor entity.

Apart from neuroendocrine tumors, radiopharmaceutical therapy has shown promising results for advanced prostate-specific membrane antigen-positive metastatic prostate cancer, with increasing evidence to support its use as an earlier line of therapy and in earlier disease stages (20–23). This shows the increasing importance of radiopharmaceutical therapy as a treatment option in a metastatic oncologic setting, highlighting the utility of cost-effectiveness analyses to determine the economic burden for the health care system and for the inclusion of such therapies in treatment guidelines.

This study found that, for our input parameters, [^177^Lu]Lu-DOTATATE was cost-efficient for the study duration at a WTP threshold of $100,000/QALY and dominant over a lifetime horizon of 20 y for retreatment with [^177^Lu]Lu-DOTATATE. When looking at the probabilistic sensitivity analyses, [^177^Lu]Lu-DOTATATE was cost-effective in 64% of Monte Carlo iterations over the trial duration. However, in total, 22.8% of iterations were dominant over the octreotide treatment. Of the deterministic sensitivity analysis variables associated with administration of [^177^Lu]Lu-DOTATATE, the number of treatment cycles and proportion of patients receiving treatment after disease progression had a strong influence on costs under the given assumptions. This was also reflected in the additional survival analysis with hazard ratios, as costs increase with higher survival times in relation to cost-efficiency; therefore, no additional change in the ICER can be recognized. Nevertheless, these changes did not result in a significant change in the ICER, specifically over the lifetime horizon, as assumptions had to be made because data were not yet available.

With regard to the significance of our analysis, the limitations mentioned below must be considered, as the PFS of both groups and the costs for octreotide and [^177^Lu]Lu-DOTATATE each had a borderline influence on the analysis, although the costs for both therapies were specified by the Centers for Medicare and Medicaid Services. Therefore, [^177^Lu]Lu-DOTATATE was a cost-effective option for patients with advanced, well-differentiated, grade 2 or 3 GEP-NETs under given conditions, but long-term data from the NETTER-2 trial are needed to confirm this assessment.

Certain limitations remain. As observed from our sensitivity analysis, the number of [^177^Lu]Lu-DOTATATE cycles for retreatment as well as the PFS of the octreotide group had a strong effect on the ICER. Furthermore, uncertainty arises from the unavailability of specific information. First, because long-term data for OS in the NETTER-2 trial are not yet available, survival data from different sources not specifically connected to radiopharmaceutical therapy were used. However, during the trial duration, no significant difference in OS was noted between treatment groups, and the NETTER-1 trial showed no significant difference in long-term OS between groups (24). Further, base case analyses with different hazard ratios for OS showed no significant change in the analysis results. However, as OS increased in both groups, the ICER for PRRT increased because of proportionally higher postprogression costs for the PRRT group compared with the octreotide group. Second, utilities for PFS and PPS were adopted from the NETTER-1 trial, which does not reflect the study population in total, again leading to uncertainty. Third, information on postprogression therapy was not documented in the published NETTER-2 trial data. Information on costs of end-of-life care and adverse events were obtained from the literature and not connected to data from the Centers for Medicare and Medicaid Services. However, when consulting the tornado diagram (Fig. 2), both parameters had little influence on the ICER. An additional comparison with studies using Centers for Medicare and Medicaid Services data found that costs were comparable for both parameters (25*,*^26^). For reasons of simplicity and to avoid further uncertainty, we assumed therapy regimens in the postprogression groups with the abovementioned therapy. We relied on data from the NETTER-2 trial, in which the control group consisted of high-dose octreotide LAR 60 mg per month. Internationally, this regimen is not a clinical routine standard for the treatment of grade 2 or 3 GEP-NETs and therefore limits generalization of our cost-effectiveness results. On the other hand, maintenance of somatostatin analog treatment after prior administration of [^177^Lu]Lu-DOTATATE, as performed in the NETTER-2 trial, corresponds to common clinical practice, and there is evidence to support this approach after first-line treatment (27). However, data are conflicting on whether somatostatin analog maintenance is beneficial for tumor control after [^177^Lu]Lu-DOTATATE instead of chemotherapy (28*,*^29^). The ongoing STOPNET (NCT06345079) and SAUNA (NCT05701241) trials are expected to answer these questions with high-quality data. If they show no benefit of somatostatin analog maintenance on tumor control after [^177^Lu]Lu-DOTATATE therapy, the latter would be unrivaled in terms of cost-effectiveness, as costs for maintenance somatostatin analogs after [^177^Lu]Lu-DOTATATE treatment could be avoided entirely.

Clinical decision-making regarding [^177^Lu]Lu-DOTATATE must also consider the primary NET location, as different therapy options with varying toxicities exist, depending on tumor origin. Whereas few alternatives to [^177^Lu]Lu-DOTATATE exist for extrapancreatic NETs, such as everolimus or cabozantinib (off-label), chemotherapy is an established alternative treatment to [^177^Lu]Lu-DOTATATE for pancreatic NETs. The latter harbors a low but severe risk for treatment-related myeloid neoplasms, reminding clinicians that clinical treatment algorithms and individual treatment decisions need to be well-balanced—beyond matters of cost-effectiveness—when transferring [^177^Lu]Lu-DOTATATE to the first-line setting.

## CONCLUSION

[^177^Lu]Lu-DOTATATE is cost-effective as a first-line treatment for patients with grade 2 or 3, well-differentiated, advanced gastroenteropancreatic neuroendocrine tumors. [^177^Lu]Lu-DOTATATE was cost-effective during the trial duration, with an incremental QALY of 0.13 at a slightly higher cost. In the probabilistic sensitivity analysis, [^177^Lu]Lu-DOTATATE was the cost-effective strategy in 64% of Monte Carlo iterations over the trial duration. Our results facilitate the assessment of its economic burden for the health care system in view of potential future inclusion in treatment guidelines. Long-term data from the NETTER-2 trial are needed to confirm this assessment.

## DISCLOSURE

Adrien Holzgreve received funding from the German Research Foundation (545058105). Lena Unterrainer received funding from the Bavarian Cancer Research Center and the Munich Clinician Scientist Program. Matthias Brendel received funding from the German Research Foundation under Germany’s Excellence Strategy within the framework of the Munich Cluster for Systems Neurology (EXC 2145 SyNergy, ID 390857198). Adrien Holzgreve received compensation for scientific consulting from ABX advanced biochemical compounds. Lena Unterrainer received speaker fees from Novartis and Astellas Pharma and consultant fees from Telix Pharmaceuticals. Matthias Brendel received consulting and speaker honoraria from Life Molecular Imaging, GE HealthCare, and Roche and reader honoraria from Life Molecular Imaging. No other potential conflict of interest relevant to this article was reported.

## Notes

KEY POINTS **QUESTION:** Is [^177^Lu]Lu-DOTATATE cost-effective in the first-line setting compared with octreotide LAR for the treatment of patients with newly diagnosed, advanced GEP-NETs? **PERTINENT FINDINGS:** We performed a cost-effectiveness analysis based on results of the phase 3 randomized NETTER-2 trial. [^177^Lu]Lu-DOTATATE was cost-effective during the trial duration, with an incremental QALY of 0.13 at a slightly higher cost. In the probabilistic sensitivity analysis, [^177^Lu]Lu-DOTATATE was the cost-effective strategy in 64% of Monte Carlo iterations over the trial duration. **IMPLICATIONS FOR PATIENT CARE:** [^177^Lu]Lu-DOTATATE may be considered as a new standard of care in first-line therapy of patients with grade 2 or 3, well-differentiated, advanced GEP-NETs. Our results facilitate the assessment of its economic burden for the health care system in view of potential future inclusion in treatment guidelines.
